# Retraction: Microbiome-based hypothesis on Ivermectin's mechanism in COVID-19: Ivermectin feeds bifidobacteria to boost immunity

**DOI:** 10.3389/fmicb.2023.1216170

**Published:** 2023-05-11

**Authors:** Frontiers Editorial Office

The journal retracts the 11 July 2022 article “Microbiome-based hypothesis on Ivermectin's mechanism in COVID-19: Ivermectin feeds bifidobacteria to boost immunity.”

Following publication, concerns were raised regarding the scientific validity of the article. An investigation was conducted in accordance with Frontiers' policies. It was found that the complaints were valid and that the article does not meet the standards of editorial and scientific soundness for Frontiers in Microbiology; therefore, the article has been retracted. This retraction was approved by the Chief Editors of Frontiers in Microbiology and the Chief Executive Editor of Frontiers.

The author has not agreed to the retraction.

